# National Cancer Survivors Conference — June 18–20, 2014

**Published:** 2014-06-13

Each year on National Cancer Survivors Day (held June 1 this year), CDC and its partners celebrate advances in cancer survivorship and reflect on the challenges facing approximately 13.4 million cancer survivors nationally. This year, CDC’s Division of Cancer Prevention and Control Survivorship Workgroup celebrates its 10th anniversary of public health work in cancer survivorship through research, surveillance, programs, systems, and environmental changes. CDC also conducts economic research to understand cancer survivorship and its impact on medical costs, out-of-pocket costs, lost productivity, employment, health insurance, and access to care (1–3)*.*

To promote cancer survivorship as a growing public health concern, CDC is cosponsoring the 7th Biennial Cancer Survivorship Research Conference, “Advancing Survivorship Care Through Multilevel Collaborations,” June 18–20, 2014, in Atlanta, Georgia (http://www.cancer.org/subsites/survivorship2014).

CDC supports states and tribal organizations in setting goals for survivorship in their comprehensive cancer control plans. The National Cancer Survivorship Resource Center (http://www.cancer.org/survivorshipcenter) also provides cancer survivorship materials that promote healthy behaviors to reduce the effects of cancer and its treatment. Additional information is available at http://www.cdc.gov/cancer/survivorship.
